# Circulating microRNAs as biomarkers for diffuse myocardial fibrosis in patients with hypertrophic cardiomyopathy

**DOI:** 10.1186/s12967-015-0672-0

**Published:** 2015-09-24

**Authors:** Lu Fang, Andris H. Ellims, Xiao-lei Moore, David A. White, Andrew J. Taylor, Jaye Chin-Dusting, Anthony M. Dart

**Affiliations:** Baker IDI Heart and Diabetes Institute, 75 Commercial Road, Melbourne, VIC 3004 Australia; Department of Cardiovascular Medicine, Alfred Heart Centre, The Alfred Hospital, 55 Commercial Road, Melbourne, VIC 3004 Australia; Monash University, Melbourne, Australia

**Keywords:** microRNAs, Myocardial fibrosis, Hypertrophic cardiomyopathy, Cardiac magnetic resonance imaging, Postcontrast T_1_ mapping

## Abstract

**Background:**

Circulating microRNAs may represent novel markers for cardiovascular diseases. We evaluated whether circulating miRNAs served as potential biomarkers for diffuse myocardial fibrosis in patients with hypertrophic cardiomyopathy (HCM).

**Methods:**

Cardiac magnetic resonance imaging with postcontrast T_1_ mapping was performed to non-invasively quantify diffuse myocardial fibrosis in HCM patients who were classified into two groups (T_1_ < 470 ms or T_1_ ≥ 470 ms, as likely or unlikely to have diffuse fibrosis, respectively). First, we screened 84 miRNAs using human serum/plasma miRNA array on plasma of 8 HCM patients (4/group based on T_1_ time) and 4 healthy controls. From the results of this initial array, 16 miRNAs were selected based on their fold changes and relevance to myocardial fibrosis for further validation by Taqman real-time PCR in 55 HCM patients.

**Results:**

Among the 16 miRNAs, the expression of miR-96-5p and miR-373-3p was low. The remaining 14 (miR-18a-5p, miR-146a-5p, miR-30d-5p, miR-17-5p, miR-200a-3p, miR-19b-3p, miR-21-5p, miR-193-5p, miR-10b-5p, miR-15a-5p, miR-192-5p, miR-296-5p, miR-29a-3p, and miR-133a-3p) were upregulated in HCM patients with T_1_ < 470 ms compared with those with T_1_ ≥ 470 ms, and 11 (except miR-192-5p, miR-296-5p and miR-133a-3p) were significantly inversely correlated with postcontrast T_1_ values. Individual miRNA had moderate diagnostic value for diffuse myocardial fibrosis (AUC: 0.663–0.742), but the diagnostic value was greatly improved (AUC: 0.87) for a combination of 8 miRNAs. In comparison, circulating markers of collagen turnover did not have predictive values for diffuse myocardial fibrosis.

**Conclusions:**

These findings suggest that circulating miRNAs provide attractive candidates as putative biomarkers for diffuse myocardial fibrosis in HCM.

**Electronic supplementary material:**

The online version of this article (doi:10.1186/s12967-015-0672-0) contains supplementary material, which is available to authorized users.

## Background

Myocardial fibrosis, a hallmark of various cardiovascular diseases, contributes to heart failure, arrhythmias and sudden death [1, 2]. Hypertrophic cardiomyopathy (HCM) is the most common monogenic cardiac disease and myocardial fibrosis is a common and early feature of HCM [3], associated with the poor prognosis in HCM patients [3, 4].

Historically, myocardial fibrosis could only be definitively diagnosed with cardiac biopsies. There are currently no reliable serological biomarkers to detect myocardial fibrosis. Recent studies have introduced cardiac magnetic resonance imaging (CMR) to noninvasively diagnose myocardial fibrosis [5]. Late gadolinium enhancement (LGE) is now an established method to identify regional myocardial fibrosis [6], but it is unable to detect diffuse myocardial fibrosis [3, 6]. Postcontrast myocardial longitudinal relaxation time (T_1_) mapping is an emerging CMR technique to evaluate diffuse myocardial fibrosis [7]. A number of T_1_ mapping techniques have been shown to correlate with histologically-quantified fibrosis [7, 8]. We and others have reported reduced T_1_ times in several cardiac disease states associated with diffuse fibrosis [7, 9–13]. However, contrast-enhanced CMR is limited by high cost and low availability, and contraindicated in patients with significant renal dysfunction and implanted cardiac devices.

MicroRNAs (miRNAs) are short, noncoding RNAs of 18–25 nucleotides that posttranscriptionally control gene expression by inhibiting protein translation or inducing target mRNA destabilization. miRNAs are powerful regulators of a wide range of important cellular processes and have emerged as dominant players in cardiovascular disease [14]. Several miRNAs, particularly, miR-21, miR-29, miR-30, and miR-133, have been implicated in the control of myocardial fibrosis [15, 16]. miRNAs are also released by cells into circulation [17]. Recent studies have suggested that circulating miRNAs serve as biomarkers for cardiovascular diseases such as acute myocardial infarct, heart failure, coronary artery disease, and hypertension [18, 19]. However, whether circulating miRNAs can serve as potential biomarkers for myocardial fibrosis has not been evaluated.

In this project, we quantified diffuse myocardial fibrosis using postcontrast T_1_ mapping time. We aimed to study: (1) changes of plasma miRNAs in HCM patients with diffuse myocardial fibrosis indicated by lower T_1_ times, as compared with patients without diffuse fibrosis or healthy controls, (2) the correlations between circulating miRNA levels and postcontrast myocardial T_1_ times, and diagnostic values of circulating miRNA levels for the detection of diffuse fibrosis.

## Methods

### Study population

We recruited 55 patients referred to the Alfred CMR department for the further evaluation of asymmetric septal hypertrophy (ASH) due to HCM from March 2011 to October 2012. ASH was defined as an interventricular septum thickness of ≥15 mm with a ratio of septal-to-lateral ventricular wall thickness of ≥1.3:1.0 as measured by echocardiography, and the diagnosis of HCM required the absence of any other condition that causes the degree of hypertrophy observed [20]. Exclusion criteria included previous septal reduction therapy, coronary artery disease, atrial fibrillation, valvular heart disease, systemic hypertension, diabetes mellitus, surgery or trauma within previous 6 months, known fibrotic or inflammatory disease or cancer, and contraindications to CMR, including pacemaker and defibrillator implantation, and significant renal dysfunction (estimated glomerular filtration rate (eGFR) <30 ml/min/1.73 m^2^). This study complied with the Declaration of Helsinki and was approved by the Institutional Ethics Committee of Alfred Healthcare. Informed consent was obtained from all participants. A subset of 8 HCM patients and 4 healthy controls were selected for miRNA array and all 55 HCM patients were included for Taqman real-time PCR analysis.

### CMR

CMR was performed using a clinical 1.5-T scanner (Signa HD 1.5-T, GE Healthcare, Waukesha, Wisconsin, USA). Volumetric LV analysis was performed using the summation of disc method with a contiguous short-axis steady-state free precession pulse sequence stack. LGE was used to identify regional fibrosis using a T_1_-weighted inversion recovery gradient echo technique, while a T_1_ mapping sequence was used to non-invasively quantify diffuse myocardial fibrosis, as previously described [7, 21]. A region of interest (ROI) was drawn around the entire LV myocardium (excluding papillary muscles) to calculate postcontrast myocardial T_1_ time. In subjects with regional fibrosis detected by LGE, these areas were excluded from the ROI for the primary analysis of postcontrast myocardial T_1_ time. T_1_ times for ROIs including areas of LGE were also calculated. To account for the potential effects of glomerular filtration rate, time delay postcontrast administration, and contrast agent relaxivity on gadolinium pharmacokinetics, corrected values of T_1_ times were used to normalize postcontrast myocardial T_1_ times to a matched state (time postcontrast administration = 20 min, eGFR = 90 mL/min per 1.73 m^2^) [22]. In addition, raw postcontrast T_1_ times of the LV blood pool (blood T1 times) were calculated.

### Echocardiography

Transthoracic echocardiography with a standard clinical protocol was performed immediately prior to CMR. Diastolic function was assessed by a combination of mitral inflow pattern (E to A ratio and deceleration time) and mitral annular velocities (e′, measured at the septal and lateral aspects of the mitral annulus in the apical 4-chamber view). Additionally, mitral E/e′ (septal, lateral and mean) was chosen as an index of LV filling pressure.

### Blood sample collection

Blood samples were obtained before CMR and collected into EDTA-tubes by venepuncture. Plasma samples (10 min centrifugation at 400 g followed by a further 10 min at 600 g) were stored at −80 °C for RNA isolation (detailed below). Serum was collected from additional tube without anticoagulants for measurement of amino terminal propeptide of type I and III collagen (PINP and PIIINP) by radioimmunoassay at the Alfred Pathology Department.

### Isolation of RNA from plasma samples

Total RNA was harvested from plasma with Qiazol lysis reagent and miRNeasy mini kit (Qiagen) according to the manufacturers’ instructions. 2 ml plasma was aliquoted into 5 eppendorf tubes (400 µl/tube). 400 µl plasma was mixed with 1 ml Qiazol lysis reagent, incubated for 5 min, and subsequently mixed with 200 µl cholorform for 3 min. After spin, the aqueous phase containing RNA was carefully collected and mixed with 100 % ethanol. RNA was purified by a miRNeasy mini spin column, and eluted by the addition of 30 µl RNase-free water. Concentration of RNA was measured by nanodrop, and RNA was stored at −80 °C for further processing.

### miScript II RT kit and miScript miRNA PCR array

0.5 µg total RNA was reversely transcribed into cDNA using the miScript II RT kit (Qiagen, Netherlands). Then, cDNA was mixed with miScript SyBR green qPCR mastermix (Qiagen). 25 µl of the cocktail was aliquoted into each well of 96 well plates containing the pre-dispensed miRNA-specific assays [Human Serum/plasma miRNA PCR Array, SABiosciences (Qiagen)]. PCR and data analysis was performed on an ABI Prism 7300 system (Applied Biosystems, USA). The transcript abundance was expressed as fold change over the value of the healthy control group calculated by 2^−∆∆Ct^ method. Eight housekeeping genes including miR-39 and U6 were used. Since U6 was quite consistent among the three groups among eight housekeeping genes, U6 was chosen as the reference miRNA for validation study by real-time PCR.

### Taqman real-time PCR

To confirm data from miScript miRNA PCR array, we measured the expression of several dysregualated miRNAs using Taqman real-time PCR (Applied Biosystems). 5 µl RNA (10 ng RNA) was reversely transcribed into cDNA using Taqman miRNA reverse transcription kit (Applied Biosystems). PCR products were then amplified from cDNA samples using the Taqman miRNA assay together with Taqman Universal PCR master mix (Applied Biosystems). The reaction volume was 20 µl. PCR reactions were performed on an ABI Prism 7500 system (Applied Biosystems). miRNAs of each group were expressed as relative miRNA expression levels using U6 as a reference value by calculating 2^−ΔCt^ i.e. 2^−Ct (miRNA)−Ct(U6)^.

### Statistical analysis

Data were expressed as mean ± SD unless otherwise stated. SPSS 17.0 was used for statistical analysis. The normality of data was tested by Kolmogorov–Smirnov test. Chi square test was used to compare discrete variables among groups. For miRNA array data, to compare differences among 3 groups, one-way ANOVA followed by Tukey multiple comparison test and Kruskal–Wallis test were used for parametic and nonparametic data, respectively. Student t test or Mann–Whitney U test was employed for comparison between the two subgroups of 55 HCM patients when appropriate. Spearman correlation coefficients were computed to assess the correlations between postcontrast T_1_ times and miRNAs. Receiver operating characteristic (ROC) curve analysis was used to calculate the area under the curve (AUC) of individual miRNA for diagnosing diffuse fibrosis. To calculate the predictive value of multiple miRNAs for diffuse fibrosis, a logistic regression model using backward stepwise (likelihood ratio) method was used to calculate predicted probabilities. Skewed data was Ln transformed prior to inclusion in the logistic regression model. AUC was then calculated using ROC curve analysis performed on predicted probabilities. A difference of *P* < 0.05 (two-sided) was considered statistically significant.

## Results

### Patient demographics

Patient demographics, CMR and echocardiograph data for miRNA array (n = 12) and real-time PCR (n = 55) are presented in Tables 1 and 2, respectively. The normal range of postcontrast myocardial T_1_ times in healthy controls in our previous study was 561 ± 47 ms [11], so T_1_ time (excluding regions of LGE) of 470 ms (approximately 2 SD below the mean of control values) was chosen to divide the HCM cohort into those likely (<470 ms) and unlikely (≥470 ms) to have significant diffuse myocardial fibrosis [21]. Postcontrast myocardial T_1_ times excluding LGE and including LGE were shown in Tables 1 and 2. T_1_ times corrected for GFR and time delay postcontrast administration were also calculated and shown. There was no significant difference in raw blood T_1_ times between groups, effectively ruling out contrast kinetics as a confounding factor for the observed differences in myocardial T_1_ time between groups. LGE (regional fibrosis) was observed in the majority of HCM patients and the mean quantity of LGE did not differ significantly between the 2 HCM groups (Tables 1, 2). For miRNA array study, lateral and mean e′ were significantly lower in HCM patients compared to controls. Lateral and mean E/e′ were significantly higher in patients with diffuse fibrosis compared to controls. As expected, septal thickness and the ratio of septal to lateral wall thickness, were significantly increased in both HCM groups compared with controls (Table 1). For real-time PCR validation study, the presence of family history was significantly higher in patients without diffuse fibrosis compared to those with diffuse fibrosis (Table 2). For both miRNA array and real-time PCR study (Tables 1, 2), there were no significant differences in age, gender, BMI, eGFR, heart rate, and blood pressure among groups. HCM groups had normal systolic function (LVEF).

**Table 1:** Subject characteristics for miRNA array

	Control	T_1_ ≥ 470	T_1_ < 470
n	4	4	4
Gender (m/f)	4/0	3/1	2/2
Age (years)	42 ± 16.3	49.5 ± 5.5	58.3 ± 9.5
Height (cm)	175.8 ± 8.6	172.8 ± 5.9	165.5 ± 11.1
Weight (kg)	77.8 ± 4.1	83.5 ± 20.3	70 ± 6.7
Body mass index (kg/m^2^)	25.4 ± 3.5	27.8 ± 5.8	25.8 ± 4
Family history of HCM (%)	NA	50 %	75 %
Resting heart beat (beats/min)	66.8 ± 9.5	69 ± 11.6	60.5 ± 7.9
Systolic blood pressure (mmHg)	120.4 ± 10.2	129.5 ± 16.6	125.3 ± 11.6
Diastolic blood pressure (mmHg)	73 ± 9.8	77.3 ± 10.8	70 ± 10.9
eGFR (mL/min/1.75 m^2^)	87.8 ± 6.5	79 ± 10.7	77.3 ± 15
Medications			
β-blockers	NA	25 %	75 %
Calcium channel blockers	NA	25 %	25 %
Angiotensin convert enzyme inhibitor	NA	0 %	0 %
Angiotensin receptor blockers	NA	50 %	0 %^†^
Echocardiography			
Left atrial volume indexed, ml/m^2^	34.2 ± 10.2	43.1 ± 10.4	56.9 ± 21.3
E/A ratio	1.53 ± 0.48	1.16 ± 0.44	1.38 ± 0.51
Deceleration time (ms)	158.5 ± 15.6	226.8 ± 44.8*	200.3 ± 25.2
Septal e′ (cm/s)	10 ± 3.6	6.2 ± 2.1	6 ± 2.2
Lateral e′ (cm/s)	13.3 ± 2.3	7.9 ± 3*	7.8 ± 2.9*
Mean e′ (cm/s)	11.7 ± 2.9	7 ± 2.5*	6.9 ± 2.3*
Septal E/e′	8.6 ± 3.4	11.1 ± 2.8	18.9 ± 8.8
Lateral E/e′	6 ± 1.3	9 ± 2.9	14.1 ± 5.9*
Mean E/e′	7.3 ± 2.3	10.1 ± 2.9	16.5 ± 7.2*
Resting LVOT gradient (mmHg)	4.5 ± 1.1	47.8 ± 68.7	51.6 ± 60
CMR			
T_1_ times (ms), excluding LGE	578.5 ± 42	561.5 ± 31.9	440.3 ± 30.3***^,††^
T_1_ times (ms), including LGE	578.5 ± 42	550.9 ± 44.6	435.3 ± 45.5***^,††^
T_1_ times (ms, corrected values)	567.6 ± 50.6	549.7 ± 34.7	422.4 ± 26.6***^,††^
Blood T_1_ times (ms)	296.0 ± 5.3	300.8 ± 31.7	302.3 ± 18.8
Septal thickness (mm)	7.75 ± 0.96	18 ± 3.6**	19 ± 3.2**
Lateral wall thickness (mm)	7.5 ± 1	8.5 ± 1.3	8.3 ± 1.7
Septal/lateral wall thickness	1.04 ± 0.07	2.12 ± 0.35**	2.35 ± 0.44**
LV mass index (g/BSA)	55.1 ± 8.9	90.6 ± 30.4	94.0 ± 28.3
LV ejection fraction (%)	57.5 ± 6.1	69 ± 2.9	67 ± 15.5
Presence of LGE (%)	NA	75 %	75 %
Quantity of LGE (% of LV) mass	NA	2.9 ± 4	10.1 ± 12.2

Data are expressed as mean ± SD

*CMR* cardiac magnetic resonance, *LV* left ventricular, *LVOT* left ventricular outflow tract, *LGE* late gadolinium enhancement, *BSA* body surface area, T_1_ times (corrected values): T_1_ times were normalized to a matched state (time post-contrast administration = 20 min, eGFR = 90 mL/min/1.73 m^2^) to account for the potential effects of glomerular filtration rate, time delay post-contrast administration, and contrast agent relaxivity on gadolinium pharmacokinetics

* *P* < 0.05, ** *P* < 0.01, *** *P* < 0.001, vs. controls, ^†^ *P* < 0.05, ^††^ *P* < 0.001, vs. T_1_ ≥ 470 ms

**Table 2:** Subject characteristics for real-time PCR

	T_1_ ≥ 470	T_1_ < 470
n	28	27
Gender (m/f)	22/6	22/5
Age (years)	49.1 ± 14.0	49.9 ± 10.8
Height (cm)	171.3 ± 10.7	174.9 ± 7.9
Weight (kg)	81.8 ± 18.7	88.5 ± 14.7
Body mass index (kg/m^2^)	27.6 ± 4.5	28.9 ± 4.0
Family history of HCM (%)	35.7 %	11.1 %*
Resting heart beat (beats/min)	63.9 ± 10.5	61.5 ± 16.1
Systolic blood pressure (mmHg)	129.7 ± 17.3	130.8 ± 14.9
Diastolic blood pressure (mmHg)	75.0 ± 9.1	71.6 ± 8.6
eGFR (mL/min/1.75 m^2^)	83.9 ± 7.6	79.7 ± 13.8
Medications		
β-blockers	50 %	63 %
Calcium channel blockers	14.3 %	29.6 %
Angiotensin convert enzyme inhibitor	10.7 %	22.2 %
Angiotensin receptor blockers	21.4 %	3.7 %
Echocardiography		
Left atrial volume indexed, ml/m^2^	48.4 ± 18.2	49.6 ± 13.9
E/A ratio	1.35 ± 0.63	1.40 ± 0.67
Deceleration time (ms)	200.0 ± 64.0	226.4 ± 44.0
Septal e′ (cm/s)	6.5 ± 1.5	5.9 ± 1.7
Lateral e′ (cm/s)	8.5 ± 1.9	7.6 ± 2.9
Mean e′ (cm/s)	7.5 ± 1.4	6.7 ± 2.1
Septal E/e′	12.5 ± 3.8	14.8 ± 5.9
Lateral E/e′	9.8 ± 3.8	12.0 ± 5.1
Mean E/e′	11.0 ± 3.4	13.4 ± 5.1
Resting LVOT gradient (mmHg)	32.8 ± 39.6	49.9 ± 51.0
CMR		
T_1_ times (ms), excluding LGE	536.3 ± 54.6	441.0 ± 30.0***
T_1_ times (ms), including LGE	521.3 ± 66.4	432.3 ± 26.2***
T_1_ times (ms, corrected values)	518.7 ± 55.2	423.8 ± 34.1***
Blood T_1_ times (ms)	310.7 ± 30.4	296.0 ± 29.3
Septal thickness (mm)	20.3 ± 5.8	19.1 ± 3.3
Lateral wall thickness (mm)	8.6 ± 1.8	9.2 ± 1.9
Septal/lateral wall thickness	2.4 ± 0.7	2.1 ± 0.5
LV mass index (g/BSA)	92.6 ± 35.0	87.4 ± 22.4
LV ejection fraction (%)	68.5 ± 7.3	70.8 ± 6.5
Presence of LGE (%)	85.7 %	81.5 %
Quantity of LGE (% of LV) mass	5.6 ± 7.5	4.7 ± 6.1

Data are expressed as mean ± SD

*CMR* cardiac magnetic resonance, *LV* left ventricular, *LVOT* left ventricular outflow tract, *LGE* late gadolinium enhancement, *BSA* body surface area, T_1_ times (corrected values): T_1_ times were normalized to a matched state (time post-contrast administration = 20 min, eGFR = 90 mL/min/1.73 m^2^) to account for the potential effects of glomerular filtration rate, time delay post-contrast administration, and contrast agent relaxivity on gadolinium pharmacokinetics

* *P* < 0.05, *** *P* < 0.001, vs. T_1_ ≥ 470 ms

### miRNA PCR array

**Fig. 1:**
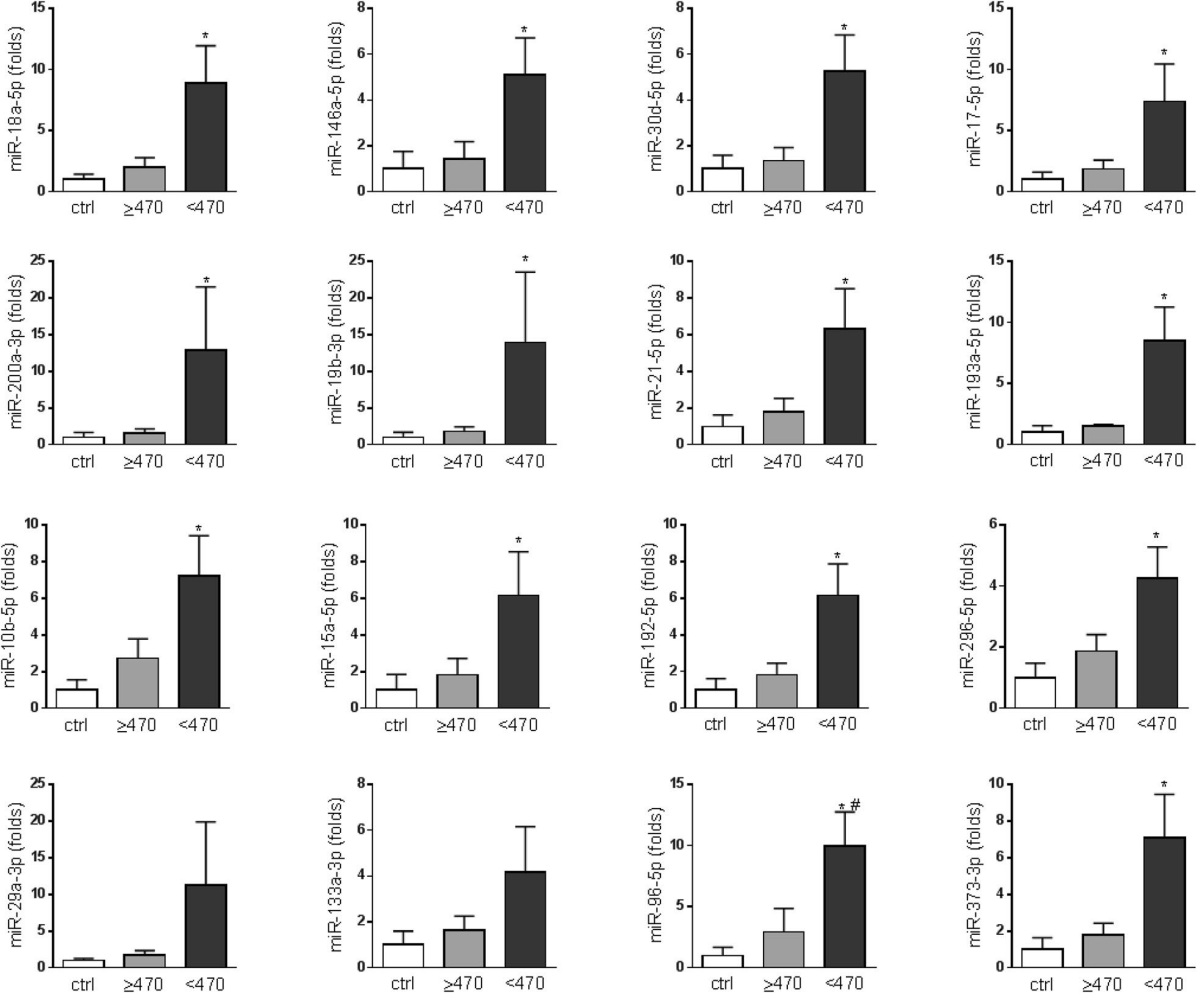
Change of plasma miRNAs in patients with lower T_1_ time identified by miRNA array. Serum/plasma miRNA array was performed on RNA samples isolated from controls, HCM patients with T_1_ ≥ 470 ms, and with T_1_ < 470 ms (n = 4/group). Data were expressed as mean ± SEM. *P < 0.05 vs. Ctrl,^ #^ *P* < 0.05 vs. T_1_ ≥ 470 ms

Human serum/plasma miRNA PCR array was performed to measure 84 miRNAs in plasma samples from 8 HCM patients and 4 controls (Additional file 1). Plasma levels of 14 miRNAs significantly differed among the 3 groups, and these 14 miRNAs also significantly increased in patients with diffuse fibrosis compared to healthy controls (all >5 folds) (Fig. 1). The upregulation in patients with diffuse fibrosis did not reach significance compared to patients without diffuse fibrosis except for miR-96-5p due to small sample size (n = 4/group). Furthermore, levels of these 14 miRNAs were negatively correlated with postcontrast T_1_ times (9 of them have r < −0.5, 5 of them have r < −0.4, Table 3). These 14 miRNAs were further validated in 55 HCM patients. Interestingly, miR-29a-3p and miR-133a-3p, known to be involved in myocardial fibrosis, were not significantly different among 3 groups (Fig. 1). We included miR-29a-3p and miR-133a-3p for further validation since their roles in myocardial fibrosis are well established.

**Table 3:** Correlations between circulating miRNAs measured by miRNA array and T_1_ times

miRNA	r	*P* value
miR-18a-5p	−0.521	0.082
miR-146a-5p	−0.658	0.020
miR-30d-5p	−0.599	0.040
miR-17-5p	−0.458	0.134
miR-200a-3p	−0.436	0.157
miR-19b-3p	−0.434	0.159
miR-21-5p	−0.443	0.150
miR-193a-5p	−0.553	0.062
miR-10b-5p	−0.548	0.065
miR-15a-5p	−0.475	0.119
miR-192-5p	−0.512	0.089
miR-296-5p	−0.557	0.060
miR-96-5p	−0.579	0.049
miR-373-3p	−0.517	0.085

Spearman correlation coefficients were computed to assess the correlations between postcontrast T1 times and miRNAs

### Validation of miRNA PCR array by real-time PCR

**Fig. 2:**
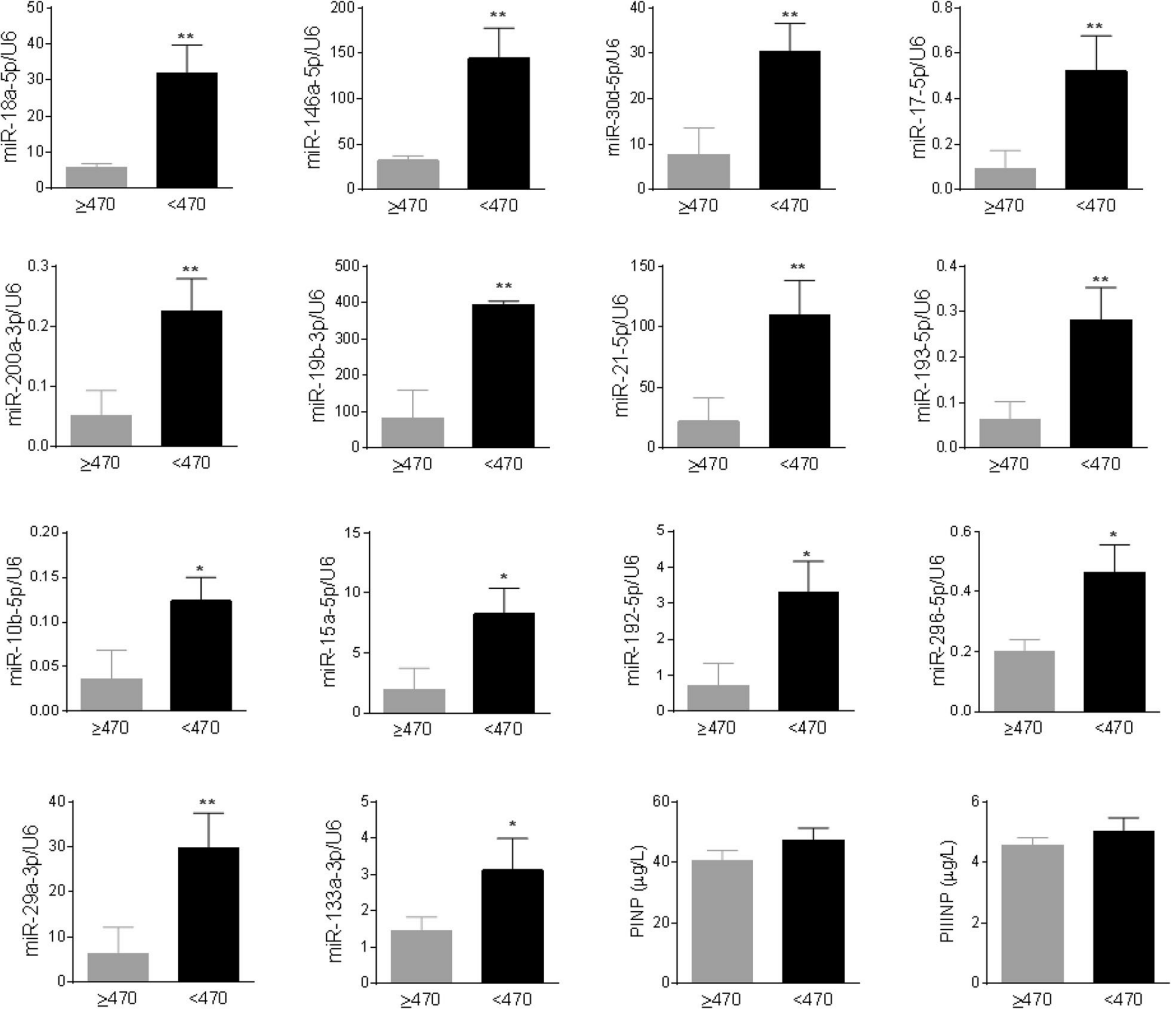
Validation of expression of miRNAs by Taqman real-time PCR. Taqman real-time PCR was performed in 55 HCM patients (n = 28 for T_1_ ≥ 470 ms and n = 27 for T_1_ < 470 ms) to validate the findings from miRNA array. In addition, circulating markers of collagen turnover, aminoterminal propeptide of type I collagen (PINP) and aminoterminal propeptide of type III collagen (PIIINP) was measured. Data were expressed as mean ± SEM. *P < 0.05, **P < 0.01

**Fig. 3:**
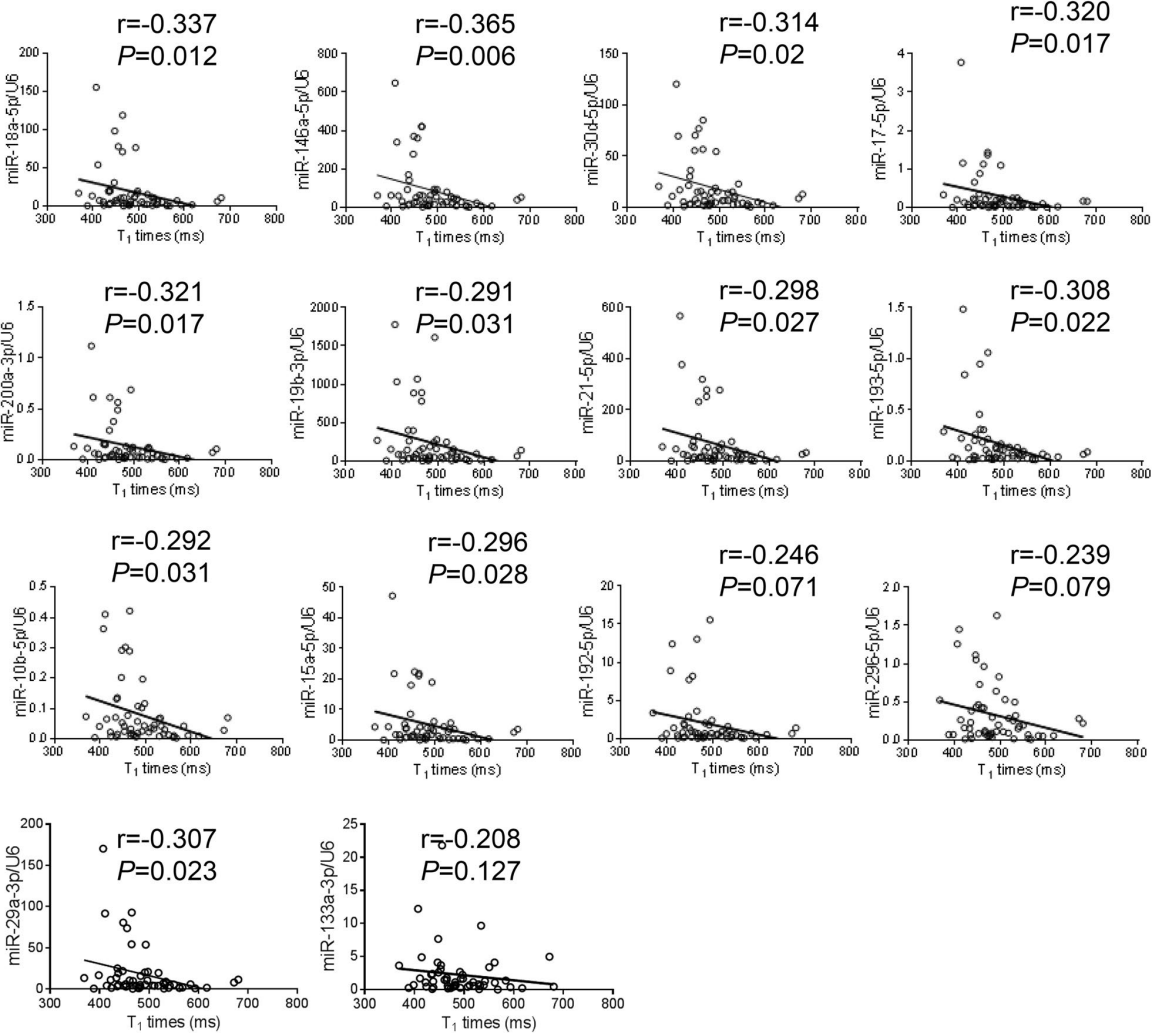
Correlations between miRNA levels and postcontrast T_1_ times. miRNA levels by real-time PCR were significantly and inversely correlated with postcontrast T_1_ times (except miR-192-5p, miR-296-5p and miR-133a) in 55 HCM patients

We validated the expression of the above 14 miRNAs plus miR-29a-3p and miR-133a-3p in all 55 HCM patients by Taqman real-time PCR. Of the 14 miRNAs that significantly differed among the 3 groups by miRNA array, 12 miRNAs were confirmed to be significantly upregulated in patients with diffuse fibrosis compared with those without diffuse fibrosis (Fig. 2). Notably, the expression of miR-96-5p and miR-373-3p was very low (data not shown). miR-29a-3p and miR-133a-3p were also significantly increased in patients with diffuse fibrosis (Fig. 2). 11 miRNAs were significantly and inversely correlated with postcontrast T_1_ times, but the inverse correlations with T_1_ times were not significant for miR-192-5p (r = 0.246, p = 0.071), miR-296-5p (r = 0.239, p = 0.079) and miR-133a-3p (r = −0.208, *P* = 0.127) (Fig. 3). Circulating miRNA levels were not correlated with the prevalence or the mean quantity of regional fibrosis quantified by LGE, or basic demographic characteristics (age, gender, BMI, blood pressure, family history of HCM, and left ventricular ejection fraction).

### Diagnostic values of plasma miRNAs for detection of diffuse myocardial fibrosis

**Fig. 4:**
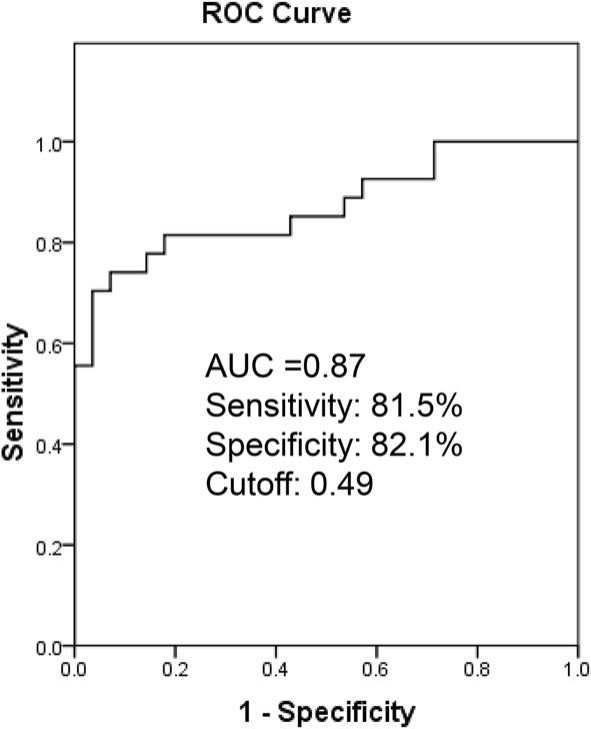
ROC analysis of the complex of 8 miRNAs (miR-18a-5p, miR-30d-5p, miR-21-5p, miR-193-5p, miR-10b-5p, miR-15a-5p, miR-296-5p, and miR-29a-3p) was used to predict diffuse fibrosis in HCM patients

The predictive power of circulating miRNAs to identify diffuse myocardial fibrosis was evaluated by ROC curve analysis. Individual ROCs of 14 miRNAs including miR-29a-3p and miR-133-3p showed moderate predictive values for the presence of diffuse myocardial fibrosis (AUC ranges from 0.663 to 0.742, Table 4). We then examined whether a complex of miRNAs had improved predictive values over single miRNA for detection of diffuse fibrosis by using logistic regression analysis. AUC for combination of all 14 miRNAs reached 0.87. To avoid overfitting of the data due to redundancies among 14 miRNAs, a logistic regression model with backward stepwise (likelihood ratio) method was employed. 8 miRNAs (miR-18a-5p, miR-30d-5p, miR-21-5p, miR-193-5p, miR-10b-5p, miR-15a-5p, miR-296-5p, and miR-29a-3p) were selected by the model and the AUC for the combination of these 8 miRNAs remained 0.87 (Fig. 4).

**Table 4:** AUC for individual miRNA

	AUC	*P* value	Cutoff	Sensitivity (%)	Specificity (%)
miR-18a-5p	0.742	0.002	6.65	70.4	71.4
miR-146a-5p	0.737	0.003	50.04	69.6	78.6
miR-30d-5p	0.729	0.004	15.19	65.2	84.4
miR-17-5p	0.722	0.005	0.16	60.9	71.9
miR-200a-3p	0.721	0.005	0.085	60.9	75
miR-19b-3p	0.712	0.007	127.38	59.3	78.6
miR-21-5p	0.710	0.007	37.18	59.3	82.1
miR-193-5p	0.709	0.008	0.11	59.3	82.1
miR-10b-5p	0.701	0.010	0.05	60.9	75
miR-15a-5p	0.694	0.013	2.88	59.3	71.4
miR-192-5p	0.681	0.021	0.72	63	75
miR-296-5p	0.681	0.021	0.26	56.5	71.4
miR-29a-3p	0.717	0.006	9.31	63	82.1
miR-133a-3p	0.663	0.038	1.42	63	78.6

Receiver operating characteristic (ROC) curve analysis was used to calculate the area under the curve (AUC) of individual miRNA for diagnosing diffuse fibrosis

### Circulating markers of collagen turnover

PINP and PIIINP are markers reflecting the status of collagen turnover. Neither PINP nor PIIINP significantly differed between patients with or without diffuse fibrosis (Fig. 2). AUC of PINP and PIIINP was 0.588 and 0.540, respectively (both *P* > 0.05).

## Discussion

Circulating miRNAs are potential biomarkers for various cardiovascular diseases [18, 19]. In the present study, we aimed to evaluate whether circulating miRNAs can serve as potential biomarkers for myocardial fibrosis in HCM patients. CMR postcontrast T_1_ mapping was used to separate HCM patients into 2 subgroups (T_1_ < 470 ms or T_1_ ≥ 470 ms as likely or unlikely to have diffuse myocardial fibrosis, respectively). We identified 14 miRNAs which significantly differed among the 3 groups (T_1_ < 470 ms, T_1_ ≥ 470 ms and controls, 4/group) using human serum/plasma miRNA array. We then validated the above 14 miRNAs plus miR-29a-3p and miR-133a-3p (known to be involved in fibrosis) using Taqman real-time PCR in 55 HCM patients. Out of 16 miRNAs selected, 14 were confirmed to be elevated in HCM patients with T_1_ < 470 ms compared to those with T_1_ ≥ 470 ms, while 11 miRNA levels significantly and inversely correlated with postcontrast T_1_ times. Furthermore, individual ROCs of these 14 miRNAs showed moderate predictive values for the presence of diffuse myocardial fibrosis (AUC ranges from 0.663 to 0.742), but a combination of miRNAs has good diagnostic value for diffuse myocardial fibrosis with an AUC of 0.87. Our results suggest that circulating miRNAs represent a novel circulating marker of diffuse myocardial fibrosis.

Myocardial fibrosis is difficult to diagnose noninvasively. Recent studies have introduced CMR to noninvasively diagnose myocardial fibrosis. However, the use of CMR is limited by its high cost and contraindications, and low availability of CMR facility and expertise. Circulating miRNAs could be practical and attractive markers for myocardial fibrosis since they are easily accessible, reliably stable and disease-specific. In the present study, we have demonstrated that circulating miRNAs are associated with diffuse fibrosis in HCM patients. Previous studies have suggested that peripheral collagen markers such as PINP and PIIIP may serve as markers for myocardial fibrosis. Circulating collagen I synthesis marker was strongly correlated with myocardial fibrosis in hypertensive patients [23], and higher circulating PIIINP concentrations were associated with increased cardiovascular mortality [24]. However, other studies did not show correlations between collagen markers (PINP, PIIINP) and myocardial fibrosis in patients with aortic stenosis [25]. Therefore, we were interested to know whether PINP and PIIINP are markers for myocardial fibrosis in patients with HCM. PINP and PIIINP did not increase in diffuse fibrosis with an AUC = 0.588 and 0.540, respectively. So, existing collagen turnover markers cannot be used in the diagnosis of diffuse fibrosis in HCM patients. In a recently published paper, the authors evaluated the correlation between circulating miRNAs and regional fibrosis quantified by LGE in HCM patients, and they found that miR-29a significantly correlated with regional fibrosis [26]. However, they did not quantified diffuse fibrosis in HCM patients. In the current study, we did not find significant correlations between miRNAs and regional fibrosis. Our study focused on the correlation of miRNAs with diffuse fibrosis and we found that 14 miRNAs including miR-29a-3p were upregulated in diffuse fibrosis. Our group has recently demonstrated that the amount of diffuse fibrosis quantified by postcontrast T_1_ mapping correlate with invasively demonstrated left ventricular stiffness in cardiac transplant recipients [27]. So, in HCM patients with CMR evidence of diffuse fibrosis, increased ventricular stiffness could contribute to diastolic heart failure.

Among 14 miRNAs identified in our study, the roles of miR-21, miR-29a, miR-30d and miR-133a in myocardial fibrosis are well established. miR-21, one of the most widely investigated miRNA, regulates fibroblast survival and promotes fibrosis through targeting sprouty/ERK pathways [28]. miR-29 is the best characterized direct regulator of extracelluar matrix protein synthesis [29], while miR-30 and miR-133a target connective tissue growth factor (CTGF) [30]. miR-17-92 cluster (miR-17, miR-18a, miR-19a, and miR-19b), also target CTGF as well as thrombospondin-1 in the context of myocardial fibrosis [31]. miR-192 and miR-200a are involved in transforming growth factor (TGF)-β signaling [32, 33]. Epithelial-to-mesenchymal transition (EMT) is another mechanism mediated by miRNAs in myocardial fibrosis and miR-10b, miR-192 and miR-200a have a role in TGF-β-dependent EMT [32–35]. miR-146a is an important regulator of the immune response and inflammation [36, 37]. In addition, miR-15a is essential for apoptosis [38], while miR-296 has been named an angiomiR [39]. However, the role of miR-193 in fibrosis is unclear. Taken together, the miRNAs identified in our study are involved in myocardial fibrosis through different mechanisms such as regulating TGF-β/CTGF signaling pathway, ECM proteins, fibroblasts and EMT.

It seems that the elevation of circulating miRNAs in HCM patients with myocardial fibrosis is caused by its upregulation in the stressed myocardium. HCM is characterized by mutations in sarcomeric proteins of cardiomyocytes. Cardiac fibroblasts are activated through the interactions between cardiomyocytes and cardiac fibroblasts. miR-21 and miR-29a are fibroblast-enriched, while miR-133a is cardiomyocyte-enriched. miR-30d comes from both cardiomyocytes and cardiac fibroblasts. Circulating miRNAs may also come from other cell types such as endothelial cells and immune cells. For example, miR-17-92 cluster are expressed on endothelial cells in addition to cardiomyocytes [40], and miR-296, miR-10b, miR-192 and miR-15a are also expressed on endothelial cells [41]. miR-146a is abundant in immune cells in addition to its expression in the heart [42] and miR-193 is also expressed on mononuclear cells [43]. It is speculated that cardiomyocytes and fibroblasts under stress may send signals to other cell types, and induce the release of miRNAs from other cells into circulation. In addition, miRNAs are potential players in such intercellular communication [19, 44] by directly acting as paracrine signals or by modulating downstream intercellular signaling mediators.

## Conclusion

We have demonstrated that 14 circulating miRNAs are associated with diffuse myocardial fibrosis quantified by postcontrast T_1_ mapping. Although individual miRNA has moderate diagnostic value for diffuse fibrosis, the diagnostic power is greatly improved (AUC: 0.87) for a combination of miRNAs. Thus, circulating miRNAs could be a favourable alternative to CMR for assessing diffuse myocardial fibrosis because of its convenience, with no contraindications.

## Additional file

**Additional file 1.** Human plasma/serum miRNA PCR array data.

## Abbreviations

HCM: hypertrophic cardiomyopathy
miR: microRNA
CMR: cardiac magnetic resonance imaging
LGE: late gadolinium enhancement
ASH: asymmetric septal hypertrophy
eGFR: estimated glomerular filtration rate
ROI: region of interest
LV: left ventricular
ROC: receiver operating characteristic
AUC: area under the curve
EMT: epithelial-to-mesenchymal transition
TGF-β: transforming growth factor-β
CTGF: connective tissue growth factor
